# Evaluation of a novel biodegradable thermosensitive keto-hydrogel for improving postoperative pain in a rat model

**DOI:** 10.1371/journal.pone.0186784

**Published:** 2017-10-23

**Authors:** Meng-Huang Wu, Ming-Hung Shih, Wei-Bin Hsu, Navneet Kumar Dubey, Wen-Fu Lee, Tsai-Yu Lin, Meng-Yow Hsieh, Chin-Fu Chen, Kuo-Ti Peng, Tsung-Jen Huang, Chung-Sheng Shi, Ren-Shyang Guo, Chang-Jhih Cai, Chiu-Yen Chung, Chung-Hang Wong

**Affiliations:** 1 Department of Orthopaedics, School of Medicine, College of Medicine, Taipei Medical University, Taipei, Taiwan; 2 Department of Orthopedics, Taipei Medical University Hospital, Taipei, Taiwan; 3 Graduate Institute of Clinical Medical Sciences, College of Medicine, Chang Gung University, Taoyuan, Taiwan; 4 Department of Anaesthesiology, Chang Gung Memorial Hospital, Chiayi, Taiwan; 5 Sports Medicine Center, Chang Gung Memorial Hospital, Chiayi, Taiwan; 6 Graduate Institute of Biomedical Materials and Tissue Engineering, College of Biomedical Engineering, Taipei Medical University, Taipei, Taiwan; 7 Department of Chemical Engineering, Tatung University, Taipei, Taiwan; 8 Biomedical Technology and Device Research Laboratories, Industrial Technology Research Institute, Hsinchu, Taiwan; 9 Institute of Biomedical Engineering, College of Engineering, College of Medicine, National Taiwan University, Taipei, Taiwan; 10 Department of Orthopaedic Surgery, Chang Gung Memorial Hospital, Chiayi, Taiwan; 11 Department of Neurosurgery, Chang Gung Memorial Hospital, Chiayi, Taiwan; 12 Department of Medical Research, Chang Gung Memorial Hospital, Chiayi, Taiwan; Kyoto Daigaku, JAPAN

## Abstract

This study evaluates the sustained analgesic effect of ketorolac-eluting thermosensitive biodegradable hydrogel in the plantar incisional pain model of the rat hind-paw. A ketorolac-embedded 2, 2'-Bis (2-oxazolin) (BOX) linking methoxy-poly(ethylene glycol) and poly(lactide-co-glycolide) (mPEG-PLGA) diblock copolymer (BOX copolymer) was synthesized as keto-hydrogel based on optimal sol-gel phase transition and in vitro drug release profile. The effect of keto-hydrogel on postoperative pain (POP) was assessed using the established plantar incisional pain model in hind-paw of rats and compared to that of ketorolac solution. Pain and sensory threshold, as well as pain scoring, were evaluated with behavioral tests by means of anesthesiometer and incapacitance apparatus, respectively. Pro-inflammatory cytokine levels (TNF-α, IL-6, VEGF, and IL-1β) around incisional wounds were measured by ELISA. Tissue histology was assessed using hematoxylin and eosin and Masson’s trichrome staining. Ten mg/mL (25 wt%) keto-hydrogel showed a sol-gel transition at 26.4°C with a 10-day sustained drug release profile *in vitro*. Compared to ketorolac solution group, the concentration of ketorolac in tissue fluid was higher in the keto-hydrogel group during the first 18 h of application. Keto-hydrogel elevated pain and sensory threshold, increased weight-bearing capacity, and significantly reduced the levels of TNF-α, IL-6, and IL-1β while enhanced VEGF in tissue fluid. Histologic analysis reveals greater epithelialization and collagen deposition around wound treated with keto-hydrogel. In conclusion, our study suggests that keto-hydrogel is an ideal compound to treat POP with a secondary gain of improved incisional wound healing.

## Introduction

Surgical wound causes a localized tissue reaction which initiates a sequence of events from the periphery and transmits centrally to result in pain [1]. Despite the advances in pain management strategies, up to 86% of patients still experience moderate-to-severe acute postoperative pain (POP) after surgery [2]. Currently, systemic narcotics and/or non-steroidal anti-inflammatory drugs (NSAIDs) are the mainstays of POP management. However, systemic administration of these medications may have adverse effects, such as narcotic-induced nausea and vomiting, respiratory depression, inhibited intestinal peristalsis and constipation, dizziness, drowsiness, skin itching, and urinary retention and NSAID-induced upper gastrointestinal ulcer bleeding and nephrotoxicity [3, 4].

The local application of anesthetics/analgesics, e.g. via cream, lotion, gel, patch, or infiltration near the surgical site can minimize these adverse effects by delivering high concentrations of drugs to the site of pain origin while lowering drug concentration systemically [5]. However, the analgesic effects of such applications have been found to be insufficient in both strength and duration, leading to questions about their overall efficacy [6, 7].

Ketorolac is a NSAID that is widely used for POP control in various surgical procedures [8–10]. However, the short-acting duration of this drug (4–6 hours) and its liquid formulation create limitations for its use as local analgesic after surgery. Thus, the development of a biodegradable carrier with sol-to-gel phase transition (i.e. aqueous at low temperatures during preparation and gel at physiologic body temperatures upon local application) may overcome the above problems [11]. In previous studies, the injectable, biodegradable and thermosensitive hydrogel has been demonstrated as a promising candidate for drug delivery [12, 13]. Based on these principles, the thermosensitive hydrogel, 2, 2'-Bis (2-oxazolin) (BOX) linking poly (ethylene glycol) monomethyl ether (mPEG) and poly (lactide-co-glycolic acid) (PLGA) diblock copolymer (BOX copolymer) developed in our study, is an easily prepared drug carrier which is also biodegradable, highly biocompatible and possess sustained drug release properties [14]. Furthermore, BOX copolymer also exhibits a broad range of gelation temperature, a slow degradation rate under physiological condition and chemical buffering systems, and low toxicity, which makes it an ideal biomaterial for delivery of ketorolac [15]. In this study, we demonstrate that ketorolac can be readily dissolved in this BOX copolymer which has a sol-to-gel phase transition between 25–45°C. When applied to surgical wounds, this ketorolac-embedded thermosensitive hydrogel (keto-hydrogel) turns viscous and enters a gel state. We further characterize the chemical properties and degradation kinetics both *in vitro* and *in vivo* as well as its efficacy in treatment of POP in rat hind-paw plantar incisional pain model.

## Materials and methods

### Chemicals

Ketorolac was purchased from Sigma-Aldrich Inc. (St. Louis, MO, US) for use in this study. The dose of ketorolac for local application in a 200+ gram rat was 1 mg (0.1 mL of 10 mg/mL) in direct application group and 1 mg (0.1 mL of 10 mg/mL) ketorolac by weight in hydrogel in our incisional pain model [16]. _D, L_-lactide and glycolide were purchased from Purac (Amsterdam, Netherlands). mPEG (number-average molecular weight, 550 g/mol) was purchased from Polyscience, Inc. (Warrington, PA, US). Stannous 2-ethylhexanoate (Sn(Oct)_2_), BOX, and succinic anhydride were obtained from Sigma-Aldrich, Inc. (St. Louis, MO, US). All chemicals used were high-performance liquid chromatography (HPLC) or extra pure grade.

### Preparation of BOX linking mPEG-PLGA diblock copolymer (BOX copolymer):

**Fig 1:**
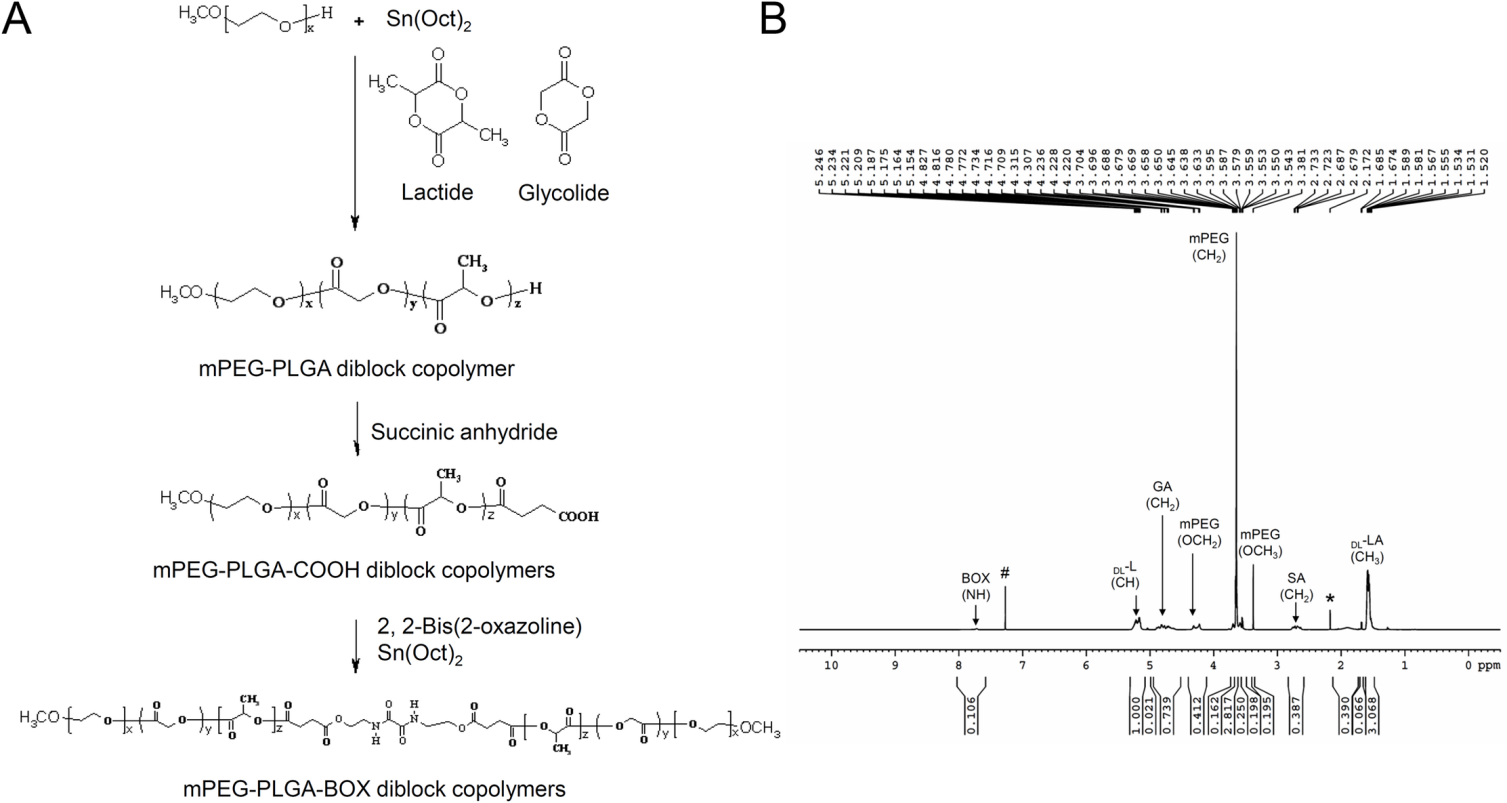
(A) Synthesis of BOX linking mPEG-PLGA diblock copolymer (BOX copolymer). (B) ^1^H NMR spectrum of mPEG-PLGA diblock copolymer (BOX copolymer). # and * denote deuterated chloroform (CDCl_3_) and H_2_O respectively.

mPEG-PLGA (550:1405) diblock copolymer was synthesized by ring-opening polymerization of monomers as previously described [14, 17]. Lactide (20 g) and glycolide (5.64 g) and mPEG (Mn, 550 g/mol, 10.04 g) in the presence of Sn(Oct)_2_ (14 μL) as the catalyst were charged into a four-neck separable reactor equipped with a stirrer, a condenser, a heater, and a thermostat. The reactor temperature was controlled by an electric heater with feedback sensor set at 160°C. After adding the catalyst, polymerization is allowed to proceed at 160°C for 8 h to obtain mPEG-PLGA diblock copolymer. Then, 1.84 g of succinic anhydride was added into the reactor and allowed to react for 4 h, after which 1.28 g of BOX was added. After the mixture completely dissolved, Sn (Oct)_2_ was added into the reactor and the linked reaction took place over the subsequent 4 h, and the product was precipitated with diethyl ether/n-hexane (1/9, v/v) to form a translucent colloid. The residual monomers were washed three times and dried in a vacuum for 24 h at 40°C to obtain BOX linking mPEG-PLGA diblock copolymer (BOX copolymer). The reaction process is shown as Fig 1A.

The molecular weight of the mPEG-PLGA diblock copolymer was determined using an Agilent GPC Addon apparatus and a RI refractive index signal detector coupled to Plgel® columns. Tetrahydrofuran was used as an eluent with a flow rate of 1 ml/min. Polystyrene standards were used for calibration. The number-average molecular weight (M_n_) and weight-average molecular weight (M_w_) of the mPEG-PLGA diblock copolymer were found to be 1492 and 2037 respectively. After linking BOX with mPEG-PLGA diblock copolymer, the M_n_ and M_w_ of the BOX copolymer were 2682 and 3915, respectively.

^1^H NMR was performed in CDCl_3_ using an NMR instrument (BRUKER DRX400) at 400 MHz at room temperature. The LA/GA ratio (78/22) was determined by integration of the signals pertaining to each monomer, such as the chemical shifts of the peaks appearing at 5.2 ppm and 1.5 ppm for the CH and CH_3_ groups of lactide respectively, and appearing at 3.6 ppm and at 4.8 ppm for the CH_2_ groups of ethylene glycol and glycolide respectively [14, 17]. But after linking BOX with mPEG-PLGA-SA diblock copolymer, the chemical shift at 3.0 ppm disappeared and characteristic peaks appeared at 3.3 ppm, 4.3 ppm, and 7.7 ppm assigned to -CH_2_CH_2_NHCO- of BOX (Fig 1B). This result conforms to the previous study [18].

### Synthesis of keto-hydrogel

BOX copolymer was synthesized by ring-opening polymerization of monomers as described above. Various amounts of ketorolac were added to 15 wt%, 20 wt%, 25 wt%, and 30 wt% copolymer aqueous solution and homogenized with vortex mixer for 1 min at or below room temperature. The final concentrations of ketorolac in copolymer solution were 10, 20, and 30 mg/mL.

### Determination of sol-gel phase transition by tube-inversion test and rheometer

To determine the sol-to-gel kinetics of the keto-hydrogel under different temperatures, a tube-inversion test at 37°C was conducted. The aqueous solutions of keto-hydrogel in various concentrations were prepared in deionized water (4°C) at concentrations between 15 and 30 wt%. The keto-hydrogel showed high solubility in water at concentrations up to 30 wt%, and all displayed a translucent emulsion solution at 4°C. After equilibration at 4°C overnight, copolymer solutions were immersed in a water bath equilibrated at various temperatures ranging from 5 to 60°C. The sol-to-gel transition and rheological properties of the keto-hydrogel were measured using rheometer (HAAKE Rheostress RS600, Thermo Fisher Scientific, Waltham, MA, US) with temperature controller (TC501) at 5–60°C. The 0.5 mL polymeric solutions were stored in the corn-plate instrument with the temperature controller and subjected to viscosity analysis. Further, the storage modulus (G’) and loss modulus (G”) were assessed.

### *In vitro* drug elution analysis of keto-hydrogel

The ketorolac concentration in each keto-hydrogel was determined by reversed-phase HPLC. To analyse the ketorolac release from BOX copolymers of various concentrations (20 wt%, 25 wt%, and 30 wt%), 1 mL of the keto-hydrogel (10 mg/mL ketorolac in 20 wt%, 25 wt%, and 30 wt% BOX copolymers) was loaded into the bottom of the 10 mL cell releasate and kept at 37°C for 5 min for gelation. 9 mL PBS was added into the cell releasate as release medium and maintained at 37°C in a thermostatic water bath with shaking at 50 rpm. At scheduled time points, 1 mL PBS media containing ketorolac was collected and the concentration of ketorolac was quantified by HPLC. One mL fresh PBS was added back to the cell releasate to maintain a total of 10 mL in the release cell. A ten-fold dilution of the sample solution was filtered through organic membrane with 0.2 mm pores and then applied to a HPLC system (HP1100, Agilent Tech., CA, US). A reversed-phase column (Zorbax SB-C18, 4.6 mm ID x 250 mm column, Agilent Tech., CA, US) was used to separate ketorolac and the mobile phase component containing acetonitrile/water (3:7, v/v). The flow rate was 0.8 mL/min while absorption was monitored at 317 nm. The concentration of released ketorolac was measured against a standard curve. All samples were assayed in triplicate.

### Animals

All animal experimental procedures were approved by the Institutional Animal Care and Use Committee (IACUC) of the laboratory animal center, department of medical research and development, Chang Gung Memorial Hospital at Chiayi (NO. 2010051101). One hundred and fourteen adult male Sprague-Dawley rats weighing 150 g were obtained from BioLASCO Taiwan Co. (Taipei, Taiwan). Rats were acclimated to laboratory environment for 2 to 3 weeks prior to experimentation and until weighing >200g. Rats were housed 3 per cage in a temperature-controlled (22°C ± 2°C) room on a 12:12-h light-dark cycle (lights on at 07:00). Food and water were provided *ad libitum*. Table 1 shows the number of animals tested in sham, control, ketorolac, keto-hydrogel and hydrogel group among various further experimental methods.

**Table 1:** Number of tested animals in sham, control, ketorolac, keto-hydrogel and hydrogel group among various experimental methods.

Experiments	Sham	Control	Ketorolac	Keto-hydrogel	Hydrogel	Sum (n = 114)
*In vivo* drug release profile	0	0	3	3	0	6
Behavioral testing	0	8	8	8	8	32
Cytokine level assay	D3: 5 D7: 5	D3: 5 D7: 5	D3: 5 D7: 5	D3: 5 D7: 5	D3: 5 D7: 5	50
Histological analysis*	D3: 1 D7: 1	D3: 3 D7: 3	D3: 3 D7: 3	D3: 3 D7: 3	D3: 3 D7: 3	26

D3: Day 3, D7: Day 7

* Histochemical analysis was evaluated by incision in both hind-paws in one rat

### Establishment of postoperative pain model

The experimental procedure for incisional pain model was followed as described by T.J. Brennan’s study [16]. All rats were anesthetized with 2% isoflurane delivered via a nose cone. The plantar aspect of right hind-paw was sterilized with 10% povidone-iodine solution, and a 1 cm longitudinal incision was made with a No. 11 blade through the skin and fascia on the plantar aspect of the foot, starting 0.5 cm from the proximal edge of the heel and extending toward the toes. The plantaris muscle was elevated and cut longitudinally while leaving the muscle origin and insertion points intact. After hemostasis with gentle pressure, either 0.1 mL sterile ketorolac (10 mg/mL) in BOX copolymer (25 wt%), 0.1 mL BOX copolymer (25 wt%), 0.1 mL of 10 mg/mL ketorolac solution, or 0.1 mL normal saline (as control) was applied to the incisional wound. Skin closure was performed with three mattress sutures using 5–0 nylon on FS-2 needle, and the wound site was covered with a triple antibiotic ointment (polymyxin B, neomycin, and bacitracin). After surgery, the animals were returned to their cages for recovery. Postoperatively, the animals were housed 3 per cage with sterile bedding made up of organic cellulose fibre. Surgical wounds were checked daily and all animals had good wound healing at the end of the study. At the end of the protocol, all animals were euthanized via inhaled CO_2_. The hind-paws were harvested for ELISA and histologic analyses.

### Determination of drug release *in vivo*

For the *in vivo* drug release studies, approximately 10 to 20 μL of tissue fluid were aspirated from around hind-paw incisional wound at various time points (6, 12, 18h, 1, 2 days) from ketorolac solution and keto-hydrogel-treated groups (n = 3). The samples were diluted in methanol (1:4) and centrifuged to obtained supernatant for drug concentration analysis using QTRAP® 5500 LC/MS/MS System (AB Sciex, Framingham, MA, US) [19].

### Behavioural testing

Behavioural tests were performed for 2 consecutive days preceding the scheduled surgery day (-D2 and -D1) and repeated once on the experimental day (D0) immediately prior to surgery (n = 8 in each group). Surgeries were only initiated when no statistical differences were observed between the basal pain parameter in these 3 days. After surgery, the pain parameters were measured at 2 h, 4 h, 6 h, (2H, 4H, 6H) and daily for 7 consecutive days thereafter (D1-7) by an operator that is blinded to the treatment.

### Assessment of mechanical hyperalgesia and tactile allodynia

Mechanical hyperalgesia and tactile allodynia were assessed in each animal using the nociceptive paw electronic pressure-meter test by rigid tips and von Frey anesthesiometer by supertips, respectively (Electronic von Frey System, IITC Inc. Life Science Instruments, CA, US). In a quiet room, rats were placed in acrylic cages (L-W-H: 12 x 20 x 17 cm) with wire grid floors for 15–30 min before testing. During this adaptation period, the paws were tested 3 times for hind-paw flexion reflex using a hand-held force transducer with a 0.5 mm diameter polypropylene tip (rigid tip) for mechanical hyperalgesia and 0.8 mm diameter flexible von Frey hairs (supertip) for tactile allodynia. A gradual increase in pressure was then applied between the five distal footpads using the transducer and the animals’ pain threshold was assessed based on the pressure at which paw withdrawal occurred. The experimental procedure is similar in either mechanical hyperalgesia or tactile allodynia whereby the flexible von Frey hair tip is applied perpendicularly to the five distal footpads with a 65 g maximal applied force. The stimulus was repeated on average three, but up to six, times until the animal presented two similar measurements. The animal was excluded if it did not present a consistent response.

### Pain scoring

Pain score was calculated based on postsurgical weight-bearing changes using an incapacitance apparatus (IITC Inc. Life Science Instruments, CA, US). The rats were trained to stand on their hind-paws in a box with an inclined plane (65° from horizontal). This box was placed above the incapacitance apparatus to allow for independent measurement of the weight that the animal applies on each hind-paw. An average of the weight-bearing capacity was taken over 10 consecutive measurements. The final pain score is normalized and expressed in percent of total body weight.

### Assessment of pro-inflammatory cytokines and angiogenic factor

Tissue cytokine levels were assessed in a manner similar to that described previously [20]. Briefly, rats were sacrificed on D3 and D7 (n = 5 in each group) and oval patches of full-thickness skin with 1 mm margins surrounding the hind-paw incision were collected. These samples containing approximately 12 mg tissue per paw were placed immediately into ice-cold 0.9% NaCl containing a cocktail of protease inhibitors (Complete™, Roche Applied Science, Indianapolis, IN, US), homogenized using a Polytron device (Brinkman Instruments Inc., Westbury, NY, US), and centrifuged for 10 min at 12,000x g at 4°C to remove large particles. An aliquot was subjected to protein assay (DC Protein Assay, Bio-Rad Laboratories, Hercules, CA, US) to normalize mediator levels. The pro-inflammatory cytokines including tumor necrosis factor alpha (TNF-α), interleukin (IL) 1-β, IL-6, and angiogenic vascular endothelial growth factor (VEGF) were chosen to be assayed based on previously reported results [21–23]. Samples were run in triplicate for each assay, and concentrations were calculated using Bio-Plex Manager software (Bio-Rad Laboratories, Hercules, CA, US).

### Histochemical staining and their quantification

For the histologic analysis, plantar skin samples were removed from both hind-paws [n = 3 (6 samples per group) in control, ketorolac, keto-hydrogel, and hydrogel. n = 1 (2 samples per group) in sham] on D3 and D7. The samples were fixed with 4% formaldehyde and embedded in paraffin. Consecutive sections (10 μm thickness) were stained with hematoxylin-eosin (H&E) and Masson’s trichrome (MT) staining. MT stain was performed by using the SIGMA Trichrome stain kit (HT15, Sigma-Aldrich Inc., St. Louis, MO, US) according to the manufacturer’s protocol. Tissue histology was scored by 2 blinded reviewers for degree of wound healing. Individual scores were combined and interpreted according to Table 2.

**Table 2:** Histological parameters for assessment of wound healing score. The histological numerical grading parameters used to calculate wound healing score. Number 1 to 4: H&E staining, Number 5 to 6: MT staining.

Number	Staining	Histologic parameters
1	H&E	Degree of granulation tissue (profound-1, moderate-2, scanty-3, absent-4)
2	H&E	Inflammatory infiltrate (plenty-1, moderate-2, few-3)
3	H&E	Orientation of collagen fibre (vertical-1, mixed-2, horizontal-3)
4	H&E	Pattern of collagen (reticular-1, mixed-2, fascicle-3)
5	MT	Amount of early collagen (profound-1, moderate-2, minimal-3, absent-4)
6	MT	Amount of mature collagen (profound-1, moderate-2, minimal-3)

### Statistical analysis

All statistical analyses were performed using Prism (6.0, GraphPad Software, Inc., CA, US). Data are expressed as mean ± SD. Differences between means were determined by Kruskal-Wallis test for comparisons of drug concentration, behavioural tests, cytokines levels and histological wound healing score between the treatment groups. Post hoc comparison was made with Dunn’s multiple comparisons test. *P* < 0.05 was considered statistically significant. The sample size of *in vivo* study was calculated based on the mechanical hyperalgesia pain withdrawal threshold at 2 h and D1 in local analgesics application group in the study by Spofford *et al*. using G*Power 3.1 [24, 25]. The total sample size needed to achieve 95% power was 8. The minimal animal number for comparing 4 groups to obtain E value more than 20 was 6 rats per group according to the resource equation method [26].

## Results

### Effect of ketorolac contents on the sol-to-gel behaviour of the BOX copolymer under varying temperatures

**Fig 2:**
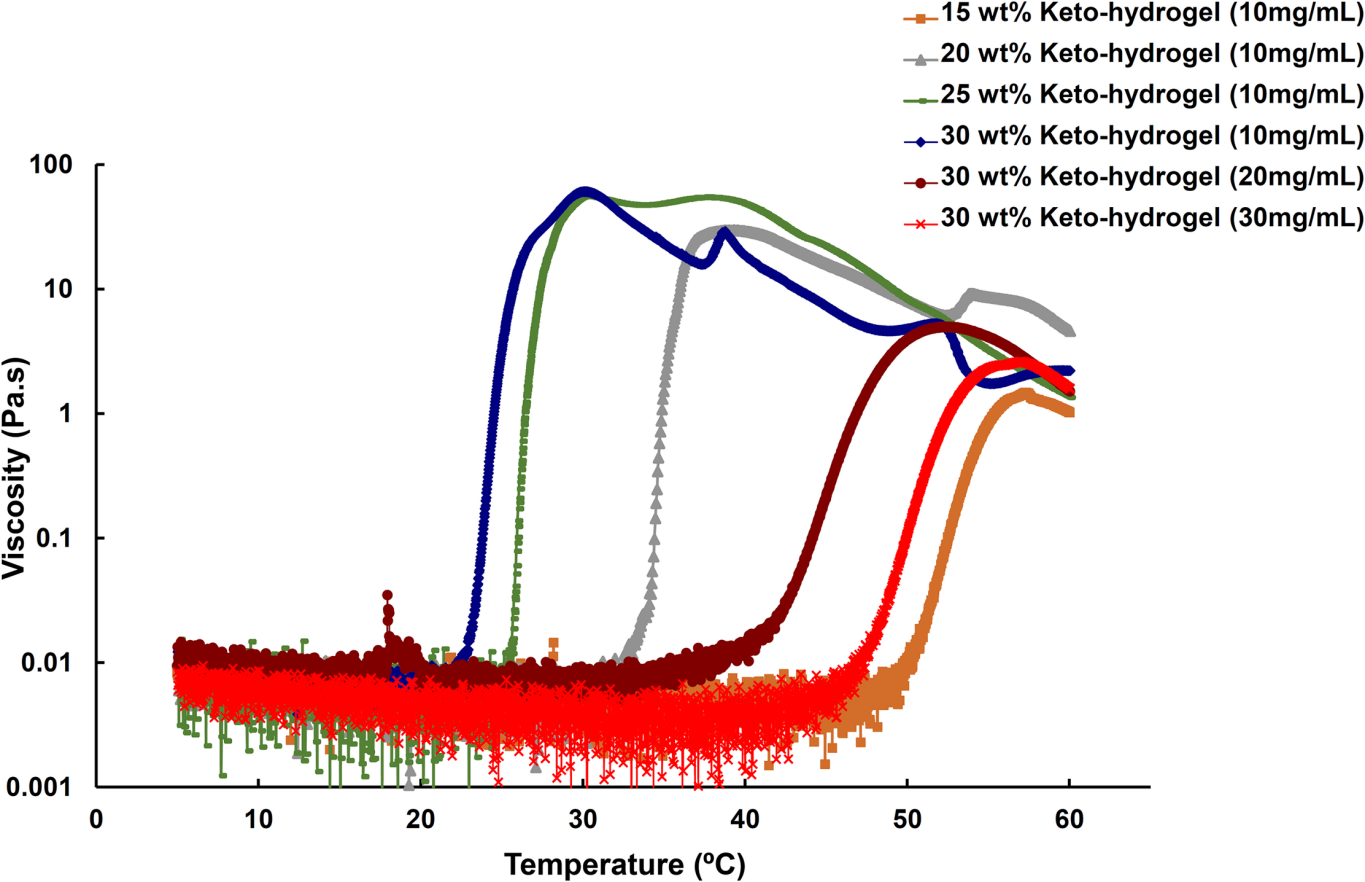
Temperature-viscosity diagram for BOX copolymer aqueous solutions of different concentrations embedded with various amounts of ketorolac from 10 mg/mL to 30 mg/mL.

**Fig 3:**
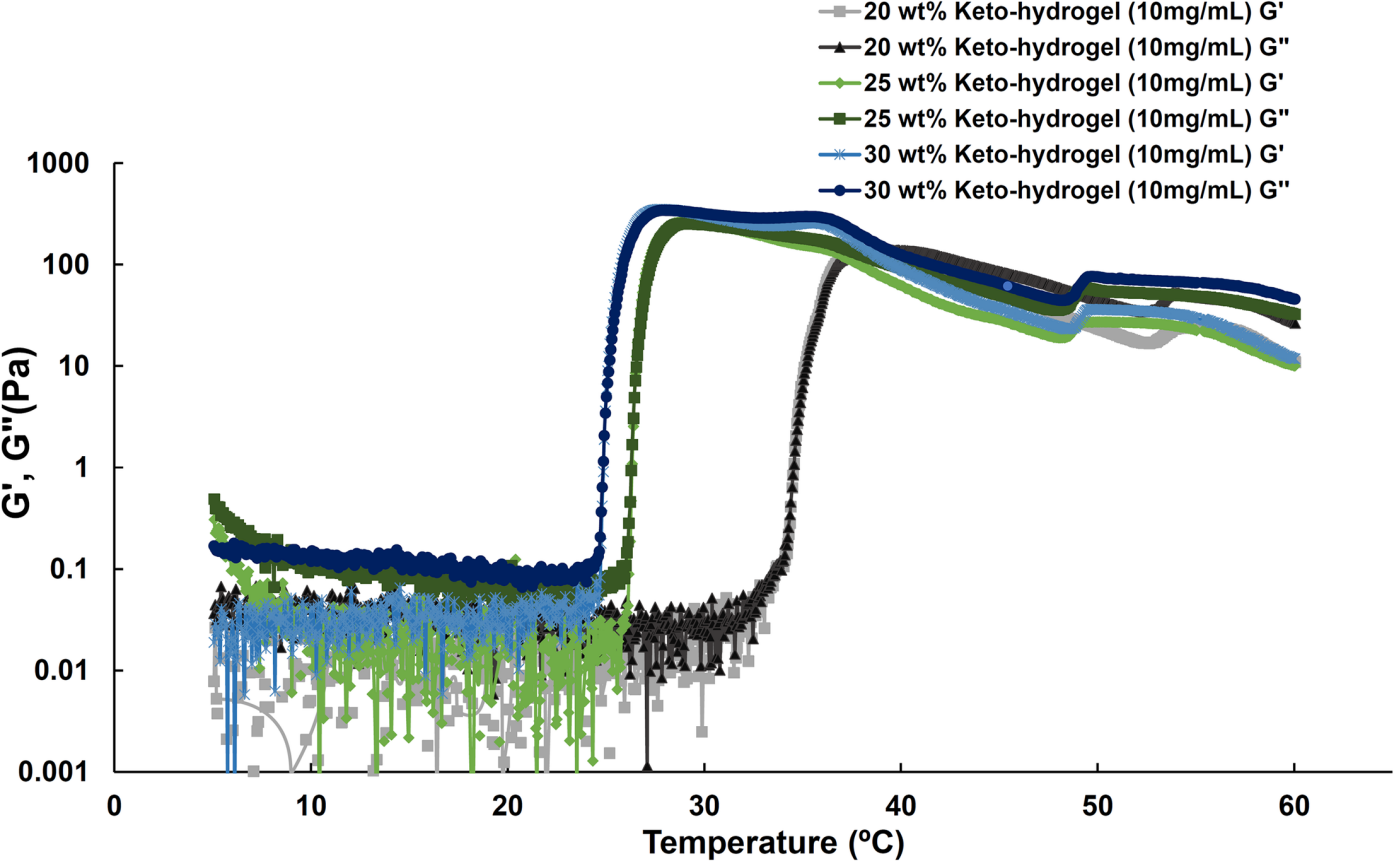
Storage modulus (G’) and loss modulus (G”) of keto-hydrogel as a function of concentration and temperature.

To determine the effect of ketorolac content on the sol-to-gel behavior of the keto-hydrogels under different temperatures, a tube inversion test was used as described previously [15]. The phase diagram of keto-hydrogels showed an inverted U-shaped curve, and the critical gelation concentration of copolymer was estimated to be 15 wt% (Fig 2). The results demonstrated that all keto-hydrogel groups exhibited sol-to-gel transitions in the temperature range between 5 and 60°C. Furthermore, the transition temperature was observed to increase with increasing ketorolac content in the copolymer solution (30 wt%). Mechanistically, the gelation temperature depends on the concentration of the polymer, the length of the hydrophobic block, and the chemical structure of the polymer: the more hydrophobic portion (BOX copolymer) leads to the larger the entropic cost of water structuring, the larger the driving force for hydrophobic aggregation, and the lower the gelation temperature [27]. Since ketorolac is water soluble and hence hydrophilic in nature, it might enhance the sol-gel temperature by water structure breaking like other salts e.g. sodium thiocyanate (NaSCN) [28]. The transition temperatures of storage modulus (G’) and loss modulus (G”) for 20 wt%, 25 wt%, and 30 wt% keto-hydrogel (all 10 mg/mL) respectively were determined as 34.6, 26.4 and 24.9°C (Fig 3). Further, the G” values were found to be higher than G’ below the transition temperature. During the phase transition, the G’ values were higher than G” (20 wt%, 34.6~36.8°C; 25 wt%, 26.4~30.7°C; 30% wt%, 24.9~29.0°C) until the gel attained the maximal viscosity. Thereafter, the G” values again became higher than G’ with progressive decline in viscosity as the temperature increased. For clinical use, the 25 wt% and 30 wt% polymer solution containing 10 mg/mL ketorolac (keto-hydrogel) exhibited suitable sol-to-gel transition temperatures that are below body temperature (Table 3).

**Table 3:** The sol-gel phase transition characteristics of keto-hydrogel by rheometer and tube inversion test at 37°C. S: solution, G: gel.

BOX copolymer	15 wt%	20 wt%	25 wt%	30 wt%	30 wt%	30 wt%
Ketorolac (mg/mL)	10	10	10	10	20	30
Sol-Gel Transition temperature (°C)	53.6	34.6	26.4	24.9	44.2	52.2
Maximal viscosity (Pa.s)	1.38	27.08	59.00	58.13	4.92	2.43
Tube inversion test at 37°C	S	G	G	G	S	S

### Ketorolac release from keto-hydrogel *in vitro and in vivo*

**Fig 4:**
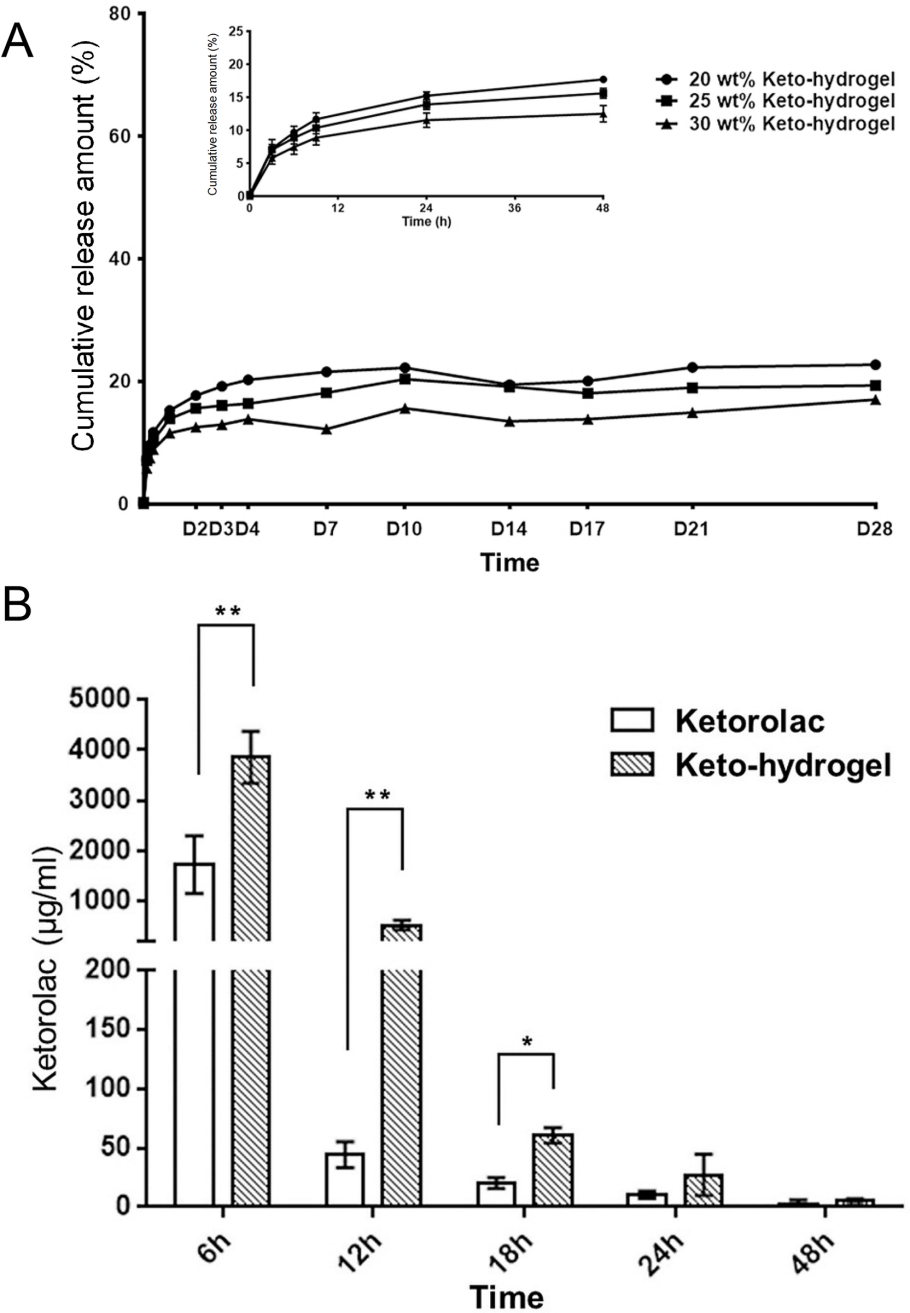
The release profile of ketorolac *in vitro* and *in vivo*. (A) *In vitro* assessment of cumulative ketorolac drug release from keto-hydrogel for 28 days. Inset shows the detail of ketorolac release for the initial 48 hours. (B) *In vivo* ketorolac concentration in tissue fluid at various time points (ketorolac solution versus keto-hydrogel; n = 3). Ketorolac concentrations are expressed as mean ± SD. *P < 0.05, **P < 0.001.

The release of ketorolac from the keto-hydrogel was assessed via *in vitro* drug elution analysis. As shown in Fig 4A, ketorolac is slowly released from 20 wt%, 25 wt%, and 30 wt% keto-hydrogel with a diffusion-controlled mechanism up to 28 days. A burst effect was observed in the release profile during the first 8 h. Both the maximum and sustained drug concentration were higher in the 20 wt% and 25 wt% keto-hydrogels (222±28.9 μg/mL and 203±13.8 μg/mL, respectively) compared to that in the 30 wt% keto-hydrogel (156±23.6 μg/mL). After 96 h, the amount of drug release appreciably reduced to 211.3 μg/mL in 20 wt% gel, 188.8 μg/mL in 25 wt% gel, and 137.9 μg/mL in 30 wt% gel.

The 25 wt% keto-hydrogel was selected as the experimental drug for *in vivo* studies for its optimal sol-to-gel transition temperature and drug release profile. Using the rat hind-paw incisional pain model, *in vivo* ketorolac concentration in tissue fluid surrounding the incision site was measured. A higher concentration of ketorolac was found in the tissue fluid of the keto-hydrogel group compared to the ketorolac solution group during the first 18 h of application, which confirmed the sustained-release property of the keto-hydrogel (Fig 4B).

### Efficacy of keto-hydrogel in hind-paw incisional pain *in vivo* model

**Fig 5:**
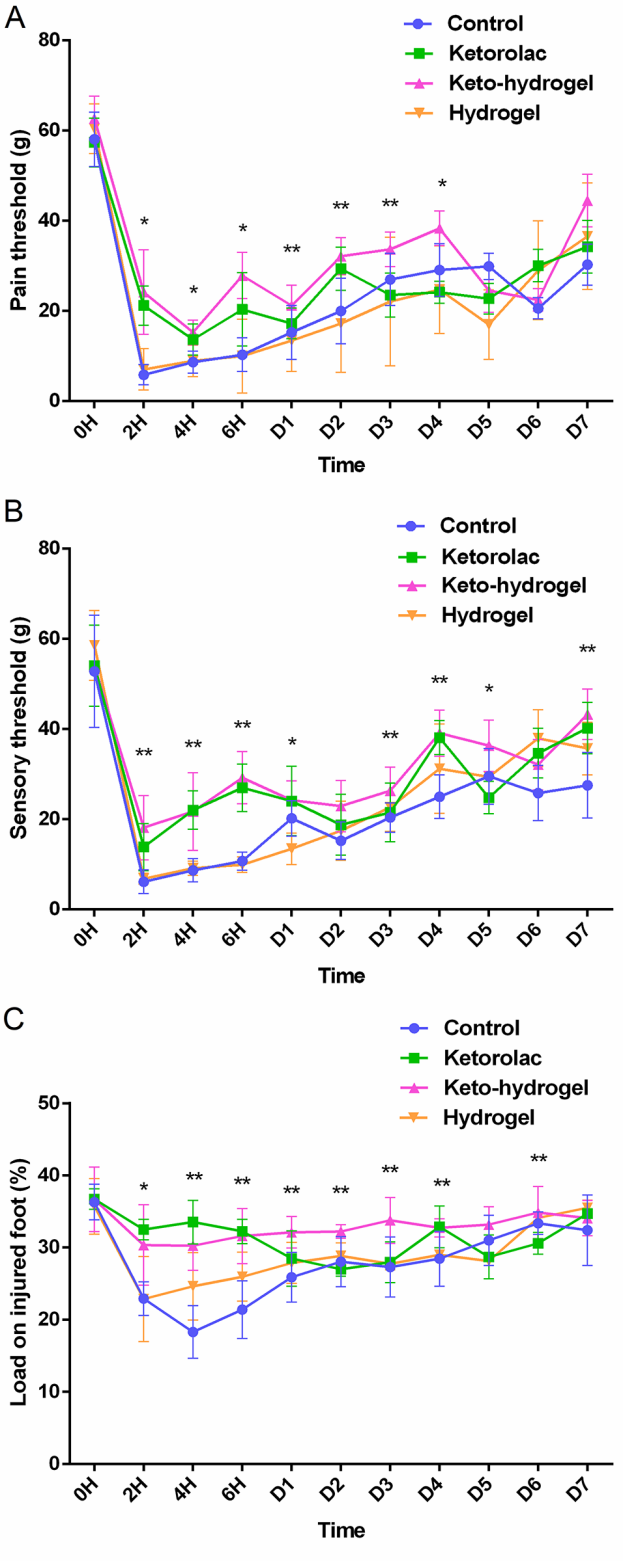
Postoperative pain and sensory assessment in rat hind-paw incisional model. (A) Pain threshold (mechanical hyperalgesia), (B) sensory threshold (tactile allodynia), and (C) pain score (relative to % body weight) of control, direct application of ketorolac solution, hydrogel, and keto-hydrogel treated rats after hind-paw incision. n = 8 per group. *P < 0.05, **P < 0.001 compared to control.

We subsequently assessed the effectiveness of 10 mg/mL keto-hydrogel (25 wt%) in reducing POP in incisional pain model. Incision of the rat hind-paw resulted in a significant decrease in pain threshold during the paw electronic pressure-meter test (Fig 5A). Application of the ketorolac solution and keto-hydrogel successfully raised the pain threshold compared to treatment with the hydrogel (25 wt% BOX copolymer) or normal saline between 2 h to D4 post surgery. The analgesic effect of keto-hydrogel treatment was similar to that of ketorolac solution during the first 4 h but was significantly greater between 6 h and D4. Evaluation of the postsurgical tactile allodynia revealed a decrease in the sensory threshold in rats receiving hydrogel or normal saline following plantar incision (Fig 5B). Application of the ketorolac solution or keto-hydrogel significantly increased sensory threshold over the course of the 2-day recovery period compared to control groups. The keto-hydrogel treatment trended toward greater effectiveness in increasing sensory threshold compared to ketorolac solution but did not reach statistical significance. Finally, using our pain scoring system, we showed that incision of the plantar surface of the hind-paw resulted in a significant reduction in weight-bearing capacity, which was ameliorated by treatment with ketorolac solution or keto-hydrogel from D1 to D4 (Fig 5C).

### Effect of keto-hydrogel on postoperative inflammation and angiogenesis in hind-paw incisional model

**Fig 6:**
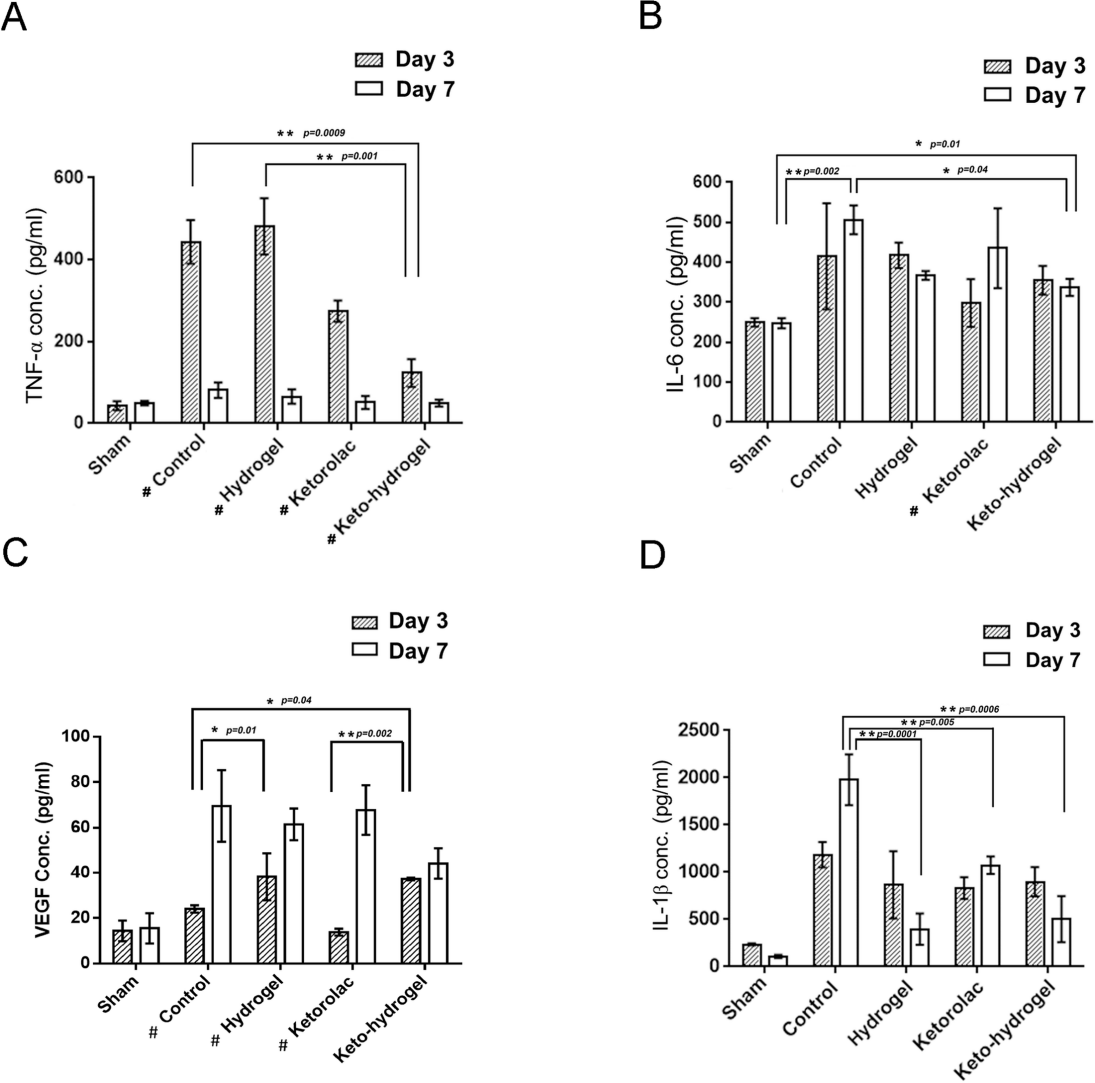
Effects of hind-paw incision on pro-inflammatory and angiogenic cytokine levels. The levels of TNF-α (A), IL-6 (B), VEGF (C), and IL-1β (D), were measured at day 3 (D3) and day 7 (D7) after incision. (N = 5 per group). Control indicates normal saline treatment, while sham indicates no surgical manipulation. * *P* < 0.05, **P < 0.001 compared between each group, # *P* < 0.05 compared between D3 and D7.

TNF-α and IL-6 are well-known inflammatory markers in acute skin incisional trauma. Compared to the sham group, the hind-paw incision in the control group resulted in increased inflammation at the surgical site as demonstrated by measured levels of TNF-α and IL-6 (Fig 6A and 6B). Among the groups, the keto-hydrogel treatment exerted greatest suppressive effect on TNF-α at D3 and returned the level of TNF-α close to baseline at D7. This reduction was also seen with ketorolac treatment although to a lesser degree. For IL-6, while at D3 the level was lowest in the ketorolac-treated group, a notable elevation was further observed from D3 to D7. A similar elevation also occurred in the control group. In contrast, for both the keto-hydrogel and hydrogel groups, the IL-6 levels reduced from D3 to D7, with the keto-hydrogel group showing the lowest level at D7.

Furthermore, to assess the effect of wound healing, the markers of angiogenesis, VEGF and pro-inflammatory phenotype macrophage marker, IL-1β were determined (Fig 6C and 6D). Our data showed that except the ketorolac-treated group, the levels of VEGF increased on D3 after skin incision, with remarkably higher levels found in both hydrogel and keto-hydrogel-treated groups compared to control. However, the level of VEGF on D7 were increased among all groups when compared to sham.

Additionally, compared to sham, all groups had increased IL-1β on D3; however, the hydrogel treatments (hydrogel-treated and keto-hydrogel treated groups) resulted in reduced level of IL-1β on D7.

### Histologic assessment of incisional hind-paw tissue

**Fig 7:**
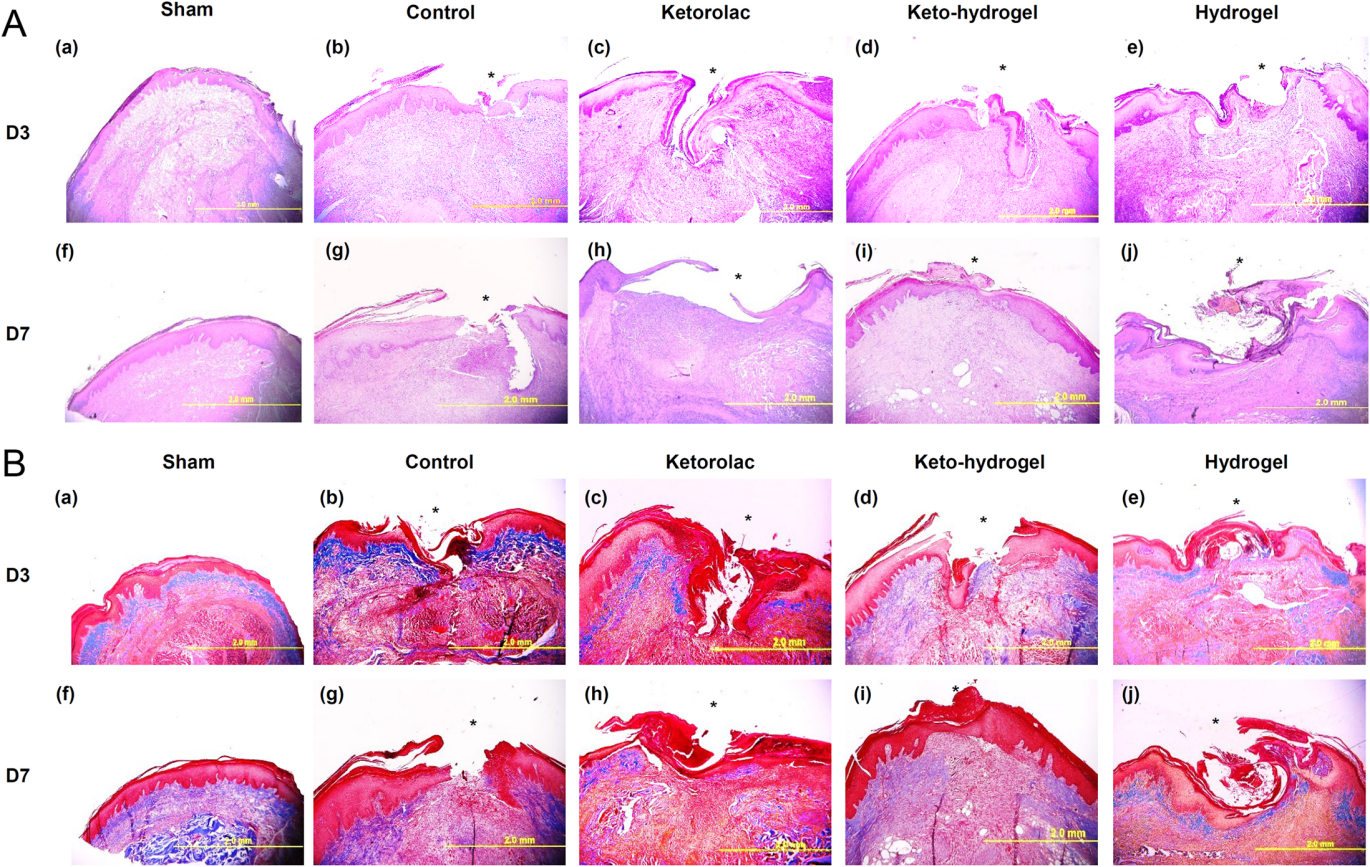
(A) Hematoxylin and Eosin (H&E) staining of the hind-paw tissue sections. Histologic sections of control, ketorolac, keto-hydrogel and hydrogel-treated hind paws after incision at D3 (a-e) and D7 (f-i). The asterisks (*) denote the surgical site. (B) Masson's trichrome stain of the hind-paw tissue section. Histologic sections of control, ketorolac, keto-hydrogel and hydrogel-treated hind-paws after incision at D3 (a-e) and D7 (f-i). Blue color is an indicative of collagenous deposition while red and pink signifies keratin and cytoplasm, respectively. The asterisks denote the surgical site.

**Fig 8:**
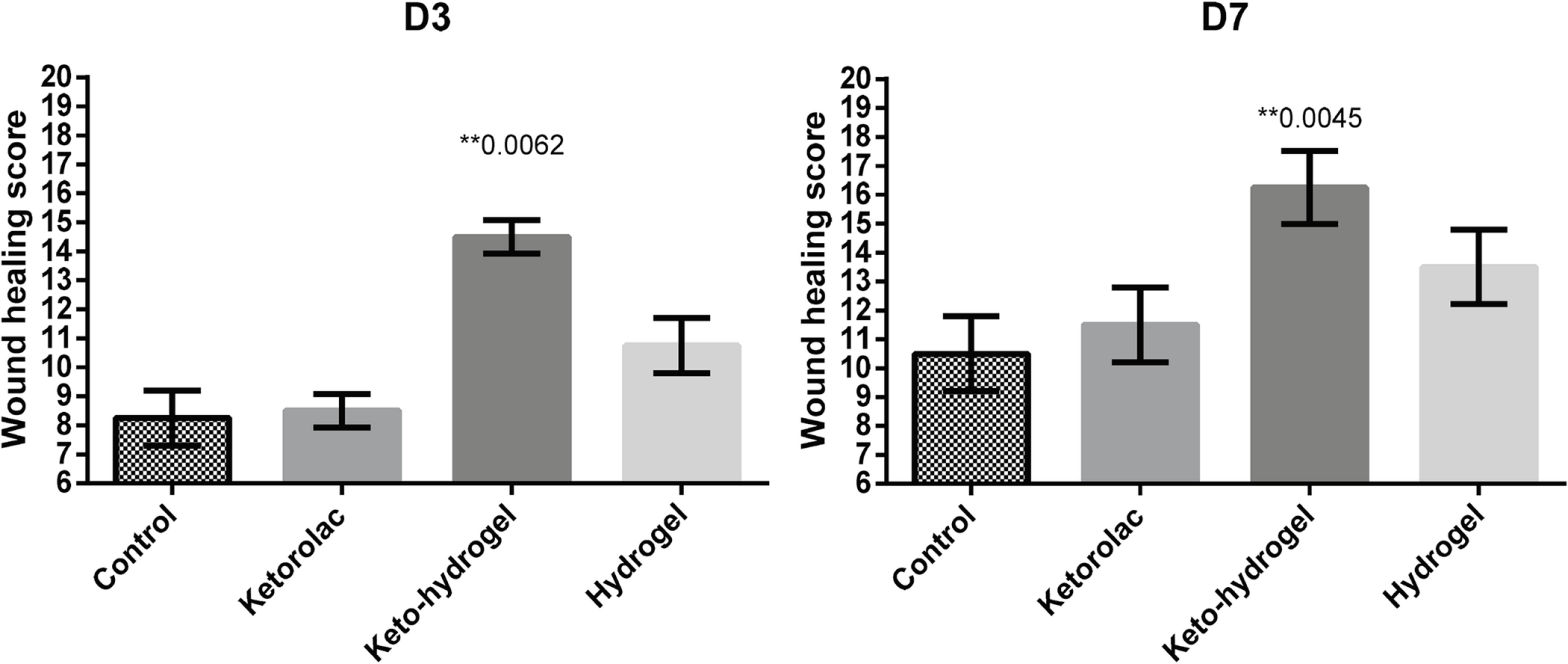
Assessment of histological grading through wound healing scores of treatment groups at D3 and D7. (n = 6 per group). ** denote p<0.01 compared to control.

H&E analysis of incisional hind-paw tissue demonstrated that compared to other groups, keto-hydrogel-treated group showed significantly rapid growth of granulation tissue, reduced inflammatory cells, and better dermal closure, particularly at D7 (Fig 7A). Furthermore, the MT staining demonstrated that keto-hydrogel-treated group revealed a marked collagenous deposition (blue color) covering the wound gap, indicating the formation of granulation tissue compared to other groups at D3 while a better matrix remodelling around incisional wound were also noticed at D7 (Fig 7B) [29]. The result of wound healing scores of each group indicated that healing in the keto-hydrogel-treated group was significantly better at both D3 and D7 when compared to other treatment groups (Fig 8).

## Discussion

To date, adequate postoperative pain control remains a challenge when attempting to balance degree and duration of analgesia with local and systemic side effects. In this study, we demonstrated that the application of a keto-hydrogel in the incisional wound of hind-paw exhibited a long-lasting analgesic effect in a rat incisional pain model. This keto-hydrogel not only is simple in design and easy to use, but also offers the benefit of a steady and sustained release of the analgesic ketorolac which further degrades into non-toxic waste products over 30 days [14].

Compared to the other biodegradable polymers, including poly (_D, L_-lactic acid) [30], poly (lactic-co-glycolic acid) (PLGA) [31], copolymers of _L_-lactide and _D, L_–lactide [32] or liposome [33], this injectable thermosensitive polymer possesses several additional advantages, including ease of preparation, high embedding efficiency of drugs or bioactive molecules, low cytotoxicity, and a formulation process that is free of harmful organic solvents. In previous studies, the BOX copolymer had been used as a carrier of bevacizumab [34], bone morphogenetic protein 2 [15], and teicoplanin [14]. The copolymer was shown to have an excellent drug-carrying capacity along with sol-to-gel phase transition which is aqueous at low temperature and turns into non-flowing gel at body temperature [14]. This property makes the compound an ideal carrier for analgesics as the aqueous phase of the copolymer at low temperatures allows an easy and even distribution of the drug.

Ketorolac is highly soluble in water and possesses significant analgesic potency [35]. In previous reports, intramuscularly administered ketorolac has been documented to be effective in a 6-hour time-frame [36]. In a study by Chou *et al*., the postoperative pain intensity at the first 3 days was the most severe [37]. In our study, ketorolac was designed to be embedded in the BOX copolymer carrier which significantly enhanced the pain and sensory threshold when compared to control, ketorolac, and hydrogel-treated groups. Importantly, a sustained analgesic response until D4 was noted following keto-hydrogel treatment, indicating better therapeutic index than currently used analgesics.

Møiniche *et al*. reported that injection of local anesthetic along an incisional wound can reduce postoperative pain [38]. Romsing and Lavand’homme *et al*. demonstrated that single dose or continuous infiltration of low-dose ketorolac to the wound resulted in significant analgesic effect [39, 40]. Furthermore, Carvalho *et al*. showed reduced concentrations of IL-6 and IL-10 in wound exudates when the incisions were treated with subcutaneous infiltration of bupivacaine or low-dose ketorolac [41, 42]. These results support the potential advantages of local applications of analgesics rather than systemic administration routes [1]. Our formulation of a keto-hydrogel was designed to include such advantages plus an even more prolonged analgesic effect due to the sustained-release nature of the hydrogel. As such, our *in vivo* data from the incisional pain experiments demonstrated that the local ketorolac concentration following single administrated dose of keto-hydrogel was significantly higher compared to ketorolac solution group during the first 18 hours. We hypothesize that the sustained analgesic effect of the keto-hydrogel contribute to an overall reduction in nociceptive inputs from the periphery and thereby decrease the central sensitization and the possibility of developing chronic pain [43].

We also analysed the indicators of inflammation TNF-α (Fig 6A) and IL-6 (Fig 6B), as well as the markers of wound healing VEGF (Fig 6C) and IL-1β (Fig 6D). TNF-α is a pro-inflammatory cytokine. In our result, at D3, keto-hydrogel exerted the greatest suppressive effect on TNF-α due to higher *in vivo* concentration of ketorolac. Notably, clustering and internalization of TNF receptors might reduce the response to TNF-α protein over time and hence does not sustain TNF-α mRNA upregulation with the passage of time [44]. This might indicate a swiftly increased TNF-α level following the initial acute inflammation phase caused by incisional injury which were further reduced during the wound healing process. Overall, the TNF-α level in all groups had returned to same level as sham group indicating that there was no prolonged acute inflammation in any group at D7.

After the skin-paw incision, the level of IL-6 at D3 in ketorolac-treated group were highly suppressed followed by keto-hydrogel group indicating their stronger anti-inflammatory actions. The notable elevated level of IL-6 from D3 to D7 indicated that the increased inflammatory activities in control and ketorolac-treated group; however, the reduced level of IL-6 from D3 to D7 in keto-hydrogel showed the anti-inflammatory effect produced by the gradual elution of ketorolac from the keto-hydrogel. Meanwhile, we also observed that the hydrogel treatment had similar decreasing pattern from D3 to D7 but not to a large extent, which might be attributed to a lesser degree of anti-inflammatory effect of hydrogel on IL-6.

The vascular endothelial growth factor (VEGF) is a well-known biomarker of angiogenesis [45]. Except the ketorolac-treated group, the level of VEGF was increased on D3 after skin incision. Notably, the VEGF levels were highest in both hydrogel and keto-hydrogel-treated groups, which reflected early angiogenesis in wound healing process [46].

IL-1β is another potent pro-inflammatory cytokine and is known to induce macrophages to become pro-inflammatory phenotype and secret more IL-1β [23]. The suppression of IL-1β can switch from pro-inflammatory to healing-associated macrophage which produce pro-healing factors. The IL-1β has also been implicated in both the induction of pain as well as in maintenance of pain in chronic states, the blockade of which might be a therapeutic target [47]. In our surprising results, the hydrogel treatment (hydrogel-treated and keto-hydrogel treated groups) resulted in reduced levels of IL-1β, indicating its effect in acceleration of wound healing and reducing pain. However, the ketorolac has no effect on blocking IL-1β which indicated insufficiency of ketorolac on the early wound healing process which is consistent with the results of VEGF. The anti-inflammatory and wound healing activity was consistent with histologic analysis showing rapid granulation tissue formation, lesser inflammatory cells, better dermal closure, more distinct aligned collagenous deposition and matrix remodelling in the keto-hydrogel treatment group, leading to improved wound healing scores. A limitation of this study is the lack of behavioral tests for responses towards heat or cold for the hyperalgesia after surgical incision [16, 24]. The testing methods for responses to heat or cold could interfere with the wound healing process, which was a principal focus in this study. Meanwhile, the improvement of withdrawal latency using locally applied analgesics had been described in the study by Spofford *et al*. which supports our study in exerting a similar analgesic effect in these behavioral tests [24].

In conclusion, this study demonstrates that keto-hydrogel is a biodegradable and thermosensitive formulation that can be effectively used in postoperative pain control. While the sustained-release nature of the hydrogel allows for delayed release of ketorolac into the incisional wound to provide prolonged analgesic effect, it also aids in overall wound healing by reducing local inflammation and enhancing angiogenesis.

## Supporting information

S1 File ARRIVE guidelines checklist. (PDF)
