# Correction: Evaluation of a novel biodegradable thermosensitive keto-hydrogel for improving postoperative pain in a rat model

**DOI:** 10.1371/journal.pone.0203372

**Published:** 2018-11-07

**Authors:** Meng-Huang Wu, Ming-Hung Shih, Wei-Bin Hsu, Navneet Kumar Dubey, Wen-Fu Lee, Tsai-Yu Lin, Meng-Yow Hsieh, Chin-Fu Chen, Kuo-Ti Peng, Tsung-Jen Huang, Chung-Sheng Shi, Ren-Shyang Guo, Chang-Jhih Cai, Chiu-Yen Chung, Chung-Hang Wong

There is an error in the Materials and methods section, under the Animals subsection. The correct IACUC approval numbers are: NO. 2010051101 and 2009121507.
